# Neuromuscular and Muscle Metabolic Functions in MELAS Before and After Resistance Training: A Case Study

**DOI:** 10.3389/fphys.2019.00503

**Published:** 2019-04-26

**Authors:** Massimo Venturelli, Federica Villa, Federico Ruzzante, Cantor Tarperi, Doriana Rudi, Chiara Milanese, Valentina Cavedon, Cristina Fonte, Alessandro Picelli, Nicola Smania, Elisa Calabria, Spyros Skafidas, Gwenael Layec, Federico Schena

**Affiliations:** ^1^Department of Neurosciences, Biomedicine and Movement Sciences, University of Verona, Verona, Italy; ^2^Department of Internal Medicine, Division of Geriatrics, The University of Utah, Salt Lake City, UT, United States; ^3^Neuromotor and Cognitive Rehabilitation Research Centre, Department of Neuroscience, Biomedicine and Movement Sciences, University of Verona, Verona, Italy; ^4^Department of Kinesiology, University of Massachusetts, Amherst MA, United States; ^5^Institute for Applied Life Sciences, University of Massachusetts, Amherst, MA, United States

**Keywords:** MELAS, exercise, neuromuscular function, muscle respiratory capacity, resistance training

## Abstract

Mitochondrial encephalomyopathy, lactic acidosis, and recurrent stroke-like episodes syndrome (MELAS) is a rare degenerative disease. Recent studies have shown that resistant training (RT) can ameliorate muscular force in mitochondrial diseases. However, the effects of RT in MELAS are unknown. The aim of this case report was to investigate the effects of RT on skeletal muscle and mitochondrial function in a 21-years old patient with MELAS. RT included 12 weeks of RT at 85% of 1 repetition maximum. Body composition (DXA), *in vivo* mitochondrial respiration capacity (mVO_2_) utilizing Near-infrared spectroscopy on the right plantar-flexor muscles, maximal voluntary torque (MVC), electrically evoked resting twitch (EET) and maximal voluntary activation (VMA) of the right leg extensors (LE) muscles were measured with the interpolated twitch technique. The participant with MELAS exhibited a marked increase in body mass (1.4 kg) and thigh muscle mass (0.3 kg). After the training period MVC (+5.5 Nm), EET (+2.1 N⋅m) and VMA (+13.1%) were ameliorated. Data of mVO_2_ revealed negligible changes in the end-exercise mVO_2_ (0.02 mM min^-1^), Δ mVO_2_ (0.09 mM min^-1^), while there was a marked amelioration in the kinetics of mVO_2_ (*τ* mVO_2_; Δ70.2 s). This is the first report of RT-induced ameliorations on skeletal muscle and mitochondrial function in MELAS. This case study suggests a preserved plasticity in the skeletal muscle of a patient with MELAS. RT appears to be an effective method to increase skeletal muscle function, and this effect is mediated by both neuromuscular and mitochondrial adaptations.

## Introduction

Mitochondrial diseases caused by mitochondrial DNA mutations are rare pathologies causing devastating physical and neural impairments (Matsumoto et al., 2005; Sproule and Kaufmann, 2008). Among these pathologies, mitochondrial encephalomyopathy, lactic acidosis, and recurrent stroke-like episodes syndrome (MELAS) is a rare neurodegenerative disease affecting several organs, particularly the nervous system and skeletal muscles with a population prevalence of 236/100000 (Matsumoto et al., 2005). The symptoms of MELAS include muscle weakness, recurrent headaches, loss of appetite, and seizures. Stroke-like episodes are common, often precipitating muscle weakness, unconsciousness, vision abnormalities and brain damage, mobility impairment, and a loss of cognitive function (Thambisetty and Newman, 2004; Henry et al., 2017). Patients with MELAS generally have a poor prognosis, as effective therapies for MELAS have yet to be found (Pfeffer et al., 2012).

Given the central role of mitochondria in energy metabolism, patients with mitochondrial dysfunctions have severe exercise intolerance (Tarnopolsky and Raha, 2005) and patients are often advised to avoid exercise, which leads to a vicious-cycle of deconditioning. Contrasting with these recommendations, recent studies have shown that aerobic exercise is actually feasible without adverse events, and even beneficial, with the prospect that this type of intervention may prevent physical deconditioning, and attenuate exercise intolerance and fatigability in these patients (Taivassalo et al., 1996, 1998, 1999; Siciliano et al., 2000). Other important achievements of the exercise training in this population are the ameliorations of whole-body aerobic capacity and muscle oxidative metabolism (Taivassalo et al., 2001; Cejudo et al., 2005; Taivassalo and Haller, 2005; Jeppesen et al., 2006). It is important to note, that besides a remarkable impairment of the aerobic metabolism, patients with MELAS also presents severe skeletal muscle losses. This imply that, perhaps, a training method that can induce both positive changes of mitochondrial (Porter et al., 2015) and neuromuscular function of skeletal muscle may provide large benefits in terms of exercise capacity and quality of life. However, to date, the research on this matter in patient with MELAS has mainly focused on the effects of very light aerobic exercise (Sproule and Kaufmann, 2008), and limited data are available on other training approaches.

Yet, some studies have reported specific positive effects of resistant training (RT) in patients with neurological and skeletal muscle dysfunctions (Tollback et al., 1999; Voet et al., 2013). Noticeably, the studies of Taivassalo et al., 1998, 1999, 2006 and Taivassalo and Haller (2004, 2005); and the recent study of Murphy et al. (2008) reported a clear physiological rationale for the utilization of RT in patients with mitochondrial dysfunction. In this last promising study have been reported improvements of muscle strength (15–25%), and ameliorations of oxidative capacity, reflected by an increase of oxygen extraction and changes in the percentage of COX deficient (Murphy et al., 2008). However, the effects of RT on the neuromuscular function and mitochondrial oxidative capacity in MELAS are currently unknown.

This study sought to investigate the effects of RT on skeletal muscle and mitochondrial function in a 21 years old male patient with MELAS. Specifically, by studying the muscles mass, neuromuscular function of locomotor-limb and *in vivo* muscular respiratory capacity after 12 weeks of RT we tested the following hypotheses: (1) After the training period, the locomotor-limb function would be increased, (2) this amelioration in muscle function could be explained, perhaps at least in part, by the improvement of neuromuscular and structural factors, and (3) *in vivo* muscle respiratory capacity would be improved in the skeletal muscle tissue recruited during the RT.

## Case Presentation

### Participant

The participant was a 21-years-old male patient with MELAS characterized by *cytochrome c oxidase* dysfunction. At the time of study, the participant suffered of severe mobility impairment (wheelchair limited), loss of hearing, partial blindness and dysphagia. Muscle weakness and asthenia were coupled with an exacerbated fatigue. From a cardiovascular point of view the heart function was reduced leading to the necessity to implant a DDDR pacemaker. The participant’s clinical characteristics were determined by qualified medical members of the research team (Table 1). Before testing, the participant abstained from physical rehabilitation for 48 h, caffeine for 12 h, and food for 3 h, and was not taking any drugs known to impact the response to the assessment procedures. This study was carried out in accordance with the recommendations of the Declaration of Helsinki. The protocol was approved by the Department of Neuroscience Biomedicine and Movement Science (Prot 227). Caregiver of the case gave written informed consent for the case participation in the study and publication of this case report. In order to better categorize the singular data of the participants with MELAS, a group of eight healthy age- sex-matched subjects served as control group (CTRL). All CTRL subjects gave written informed consent. CTRL subjects were healthy recreationally active men, demographic characteristics are reported in Table 1.

**Table 1:** Subject characteristics.

	MELAS		CTRL
		
	PRE	POST	
Age (years)	21	21	22 ± 2
Digit Span	3	3	
Memory of Prose (Range 0–28)	Memory 4 Recall 8	Memory 4 Recall 8	
Rey Auditory Verbal Learning Test (Range 0–75)	Memory 23/75 Recall 6/15	Memory 23/75 Recall 6/15	
Clock Drawing Test (Range 0–10)	0	0	
Frontal Assessment Battery (Range 0–18)	8	8	


*Data of the control participants (CTRL) are given as mean ± standard deviation. MELAS, Mitochondrial encephalomyopathy, lactic acidosis, and recurrent stroke-like episodes syndrome.*

### Experimental Overview

The participants visited the laboratory on three occasions separated by 24 h. The first visit comprised body composition testing (DXA), and the clinical assessments. At the second visit, the participants completed a familiarization with the interpolated twitch and *in vivo* mitochondrial respiration capacity protocols. At the third visit, the participants completed an *in vivo* mitochondrial respiration capacity protocol utilizing a Near-infrared spectroscopy device (NIRS) on the right plantar-flexor muscles. After 60 min of recovery, maximal voluntary torque (MVC), electrically evoked resting twitch (EET) and maximal voluntary activation (VMA) of the right leg extensors (LE) muscles were determiner with the interpolated twitch technique. Only for the participant with MELAS these evaluations were repeated after 12 weeks of RT.

### Clinical Assessments

From a clinical point of view, the participant with MELAS showed dysphagia to solids (he had a modified diet and assumed food integrators). He presented with easy fatigability and decreased muscle mass. No limitation of (main joints) range of motion was found at the upper and lower limbs. Muscle tone was not affected in a relevant way but needed some assistance to change posture (from supine to sitting posture as well as from sitting to standing posture).

The cognitive assessment battery utilized in this study was very limited due to the severe hearing loss, vision acuity deficits and hemianopia, in addition to the easy fatigue and eyelid ptosis. The evaluation was focused on the main cognitive deficits reported in the literature in the MELAS. The patient was perfectly oriented in the space-time parameters, but with important deficits of sustained and selective attention, with an easy distractibility. The participant demonstrated deficits in short-term and long-term verbal memory. The evaluation of executive functions estimated with Frontal Assessment Battery and Clock Drawing Test (Appollonio et al., 2005) revealed a lack of planning ability, verbal ideation and inhibitory control. Tests’ scoring are reported in Table 1.

### Body Composition

Body composition (body fat and lean mass) was assessed by means of Dual energy X-ray absorptiometry using a total body scanner (QDR Explorer W, Hologic, MA, United States; fan-bean technology, software for Windows XP version 12.6.1) according to the manufacturer’s procedures. The scanner was calibrated daily against the standard supplied by the manufacturer to avoid possible baseline drift. Whole body scanning time was about 7 min. Data were analyzed using standard body region markers: upper and lower extremities, head, and trunk (pelvic triangle plus chest or abdomen). Additionally, the DXA scans were examined using non-standard body region markers to define thigh segments. The thigh region was delineated by an upper border formed by an oblique line passing through the femoral neck to the horizontal line passing through the knee (Skalsky et al., 2009). All scanning and analyses were performed by the same operator to ensure consistency. In our lab the precision error (percent coefficient of variation with repositioning) of whole-body DXA measurements is 2.3, 0.5, and 2.8% for fat mass, lean mass and percent fat mass, respectively.

### *In vivo* Mitochondrial Respiration Capacity

The assessment of *in vivo* mitochondrial respiration capacity was performed via a non-invasive approach of the muscle oxygen consumption (mVO_2_) as previously described by Ryan et al. (2013, 2014a), Adami and Rossiter (2018). NIRS data were obtained using a device (OxiplexTS, ISS, Champaign, IL, United States), equipped with a standard acquisition probe (emitter detector distances of 2.0, 2.5, 3.0, and 3.5 cm). The values of oxygenated hemoglobin (Gonzalez-Alonso et al., 2001) and deoxygenated hemoglobin [HHb] were recorded at 4 Hz and expressed in micromoles using the Beer Lambert Law and multi-distance frequency resolved spectroscopy.

The NIRS probe was positioned longitudinally on the belly of the right plantar flexor muscles. The probe was secured with double-sided adhesive tape and a Velcro strap around the calf. After 30 min of warm-up period, the NIRS device was calibrated using a phantom with known optical properties A blood pressure cuff was placed proximal to the NIRS probe around the popliteal area. The blood pressure cuff was controlled with a rapid-inflation system (Hokanson E20, D.E. Hokanson) set to a pressure of >250 mmHg and powered with an air compressor.

The NIRS experimental protocol consisted of two measurements of resting mVO_2_ after the inflation of the blood pressure cuff for 30 s. mVO_2_ was calculated as the rate of change of the HHb signal during the arterial occlusion via linear regression. Following the resting measurements, participants performed a 30 s dynamic contractions of the plantar flexors muscle to increase mVO_2_. Upon relaxation, the recovery kinetics of mVO_2_ were measured using a series of transient arterial occlusions with the following timing: 5 s on/5 s off for occlusions 1–5, 7 s on/7 s off for occlusions 6–10, and 10 s on/10 s off for occlusions 11–20. Post-exercise mVO_2_ was calculated for each occlusion using a linear regression. The mVO_2_ recovery kinetics were determined by fitting the time-dependent changes during the recovery period to a mono-exponential curve described by the following equation:

$$Y(t)=Y_{end}+Y_{amp}\left(1-e^{-\left(t-TD/\tau \right)}\right)$$

where Y_end_ is the level of variable measured at end-of-exercise and Y_res_ refers to the amplitude of the response, TD represent the time delay (TD), and τ reflects the time constant of the recovery, a relative measure of muscle oxidative capacity (Adami and Rossiter, 2018). Model variables were determined with an iterative process by minimizing the sum of squared residuals (RSS) between the fitted function and the observed values. Goodness of fit was assessed by visual inspection of the residual plot and the frequency plot distribution of the residuals, Chi square values, and the coefficient of determination (*r*^2^), which was calculated as follows:

$$r^{2}=1-\left({SS}_{reg}/{SS}_{tot}\right)$$

with SS_reg_, the sum of squares of the residuals from the fit and SS_tot_, and the sum of squares of the residuals from the mean.

### Neuromuscular Function of Locomotor-Limb

Maximal voluntary and electrically evoked muscle contractions of the LE muscles were measured utilizing a custom-made setup (Venturelli et al., 2015). Subjects were seated in an upright position with back support. The hip and the knee were flexed at 90°, and the right ankle were attached, via a strap and rigid steel bar, to a force transducer (DBBSE-100 kg, A2829. Applied Measurements Limited, Aldermaston Berkshire, United Kingdom). The output from the force transducer was amplified (INT2-L, London Electronics Limited, Sandy Bedfordshire, United Kingdom), and recorded at a sampling rate of 5 KHz with a PowerLab-16/35 data acquisition system (ADInstruments, Bella Vista, NSW, Australia).

### Voluntary and EET Normalized Force

In the participant with MELAS the determination of muscle cross sectional area via magnetic resonance imaging was not possible due to the implanted pacemaker. Therefore, LE voluntary muscle normalized force was calculated by dividing torque of the LE isometric maximal voluntary contraction (MVC), by the lean muscle mass of the corresponding muscles (nMVC) from DXA. Similarly, EET normalized force was calculated by dividing torque of the LE electrically evoked EET, by the lean muscle mass of the corresponding muscle (nEET).

VoluntaryandEETnormalizedforce=torque/thighleanmusclemass

### Electromyography

M-waves were recorded during femoral nerve stimulation in the vastus lateralis muscle (detailed in next section). Pairs of full-surface solid adhesive hydrogel electrodes (H59P, Tyco Healthcare Group, Mansfield, MA, United States) were positioned lengthwise over the muscle belly, with an inter-electrode distance (center-to-center) of 20 mm. The ground electrodes were fixed over the ipsilateral patella. Light skin abrasion followed by skin cleansing kept electrical impedance below 10 kΩ. EMG signals were amplified with a pass-band of 10 Hz–1 kHz and digitized online at a sampling frequency of 5 kHz.

### Nerve Stimulation

Each test procedure began with the determination of the maximal M-wave and EET responses in the resting LE muscle. Briefly, current intensity was progressively increased from 0 mA to the value beyond which there was no further increase in M-wave amplitude. The stimulus utilized for the study was set at the 125% of the intensity required to produce a maximal M-wave response. Electrical stimuli were delivered using circular (diameter 5.0 cm) self-adhesive electrodes (Dermatrode, American Imex, Irvine, CA, United States) positioned in the femoral triangle, 3–5 cm below the inguinal ligament, and the anode placed over the iliac crest. The EET were evoked in the passive muscle using electrical stimulation consisting of single square-wave pulses of 0.1-ms duration, delivered by a Digitimer DS7h constant-current stimulator (Digitimer Ltd., Welwyn Garden City, United Kingdom). The EET was measured 5 s after a 5 s MVC of the LE and this procedure was repeated six times. Consequently, EET was assessed in the potentiated state. The interval between the MVCs was 30 s. Peak torque, was assessed for each EET (Sandiford et al., 2005). Voluntary activation of the LE muscles during the MVCs was assessed using a superimposed twitch technique (Merton, 1954). Briefly, the force produced during a single twitch superimposed on the MVC was compared with the force produced by the electrically evoked EET produced, at rest, 5 s after the MVC.

### Exercise Resistance Training

Resistant training included 60 min three times a week of high-intensity strength training for an overall exercise duration of 12 weeks. Sessions started with 10 min of warm up which included active joint mobilization of lower and upper limbs. Then, the participant performed 3 sets of 10 reps of strength exercises at 85% of 1 repetition maximum (1RM). 1RM was determined by means of Brzycki method. Briefly, the participant executed progressive series of the isotonic exercise until the offered resistance was impossible to be sustained for 5/6 repetitions. The number of repetitions, and the relative workload, were used in the Brzycki equation. 1RM was adjusted every 2 weeks and the corresponded training load was increased. RT ended with stretching exercises for all the muscle involved in the training. All training sessions were supervised by a skilled kinesiologist.

### Data Analysis and Interpretation

Control group data and muscle function values measured in the participant with MELAS during six repetitions of isometric LE are presented as mean ± standard deviation. Due to the descriptive nature of this single case study any specific analysis was applied to the collected data.

## Results

### Characteristics of the Participants

One patient with MELAS and eight healthy controls were successfully enrolled in the study. In Table 1 are displayed demographic, clinical characteristics of the participants. The patient with MELAS attended 31 (86%) of the 36 scheduled sessions of RT and no adverse events occurred either during exercise or the recovery phase of RT.

### Muscle Mass and Function

**FIGURE 1:**
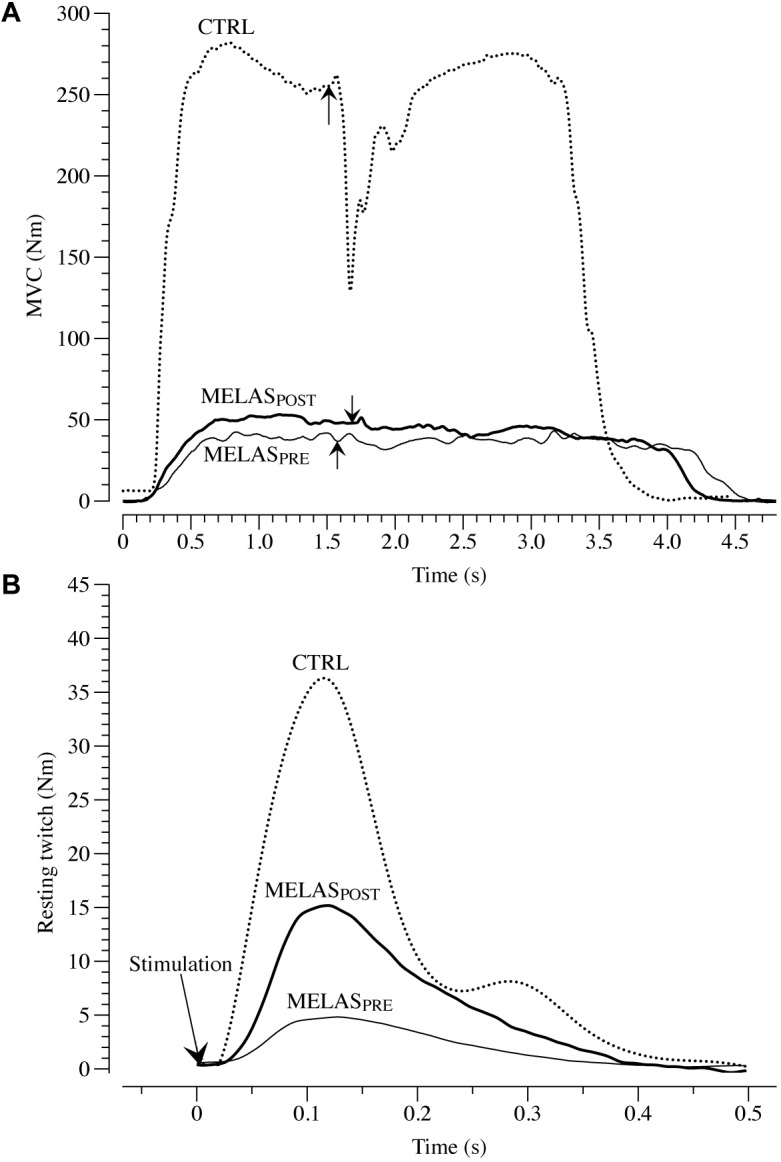
Maximal voluntary contraction (MVC) and electrical evoked resting twitch (EET) characteristics. **(A)** Presents example tracings of the superimposed twitch technique utilized to determine muscle voluntary activation in leg extensors (LE) in a patient with mitochondrial encephalomyopathy, lactic acidosis, and recurrent stroke-like episodes syndrome (MELAS). The superimposed twitches (arrows) were imposed at the highest volitional steady-state torque. Representative examples of EET torque-time curves from LE are illustrated in **(B)**. MELAS_PRE_ and MELAS_POST_ represents force-tracing in a patient with MELAS before and after 12 weeks of resistance training. Dashed line represent an example tracing of MVC **(A)** and EET **(B)** of a healthy control (CTRL).

As illustrated in Table 2 after 12 weeks of RT the participant with MELAS exhibited a marked increase in body mass (1.4 kg) and thigh muscle mass (0.3 kg) coupled with no detectable change in body fat. Representative images of superimposed twitches evoked in the participant with MELAS and a healthy control during the MVCs of LE are displayed in Figure 1A. Representative EET tracings in the LE muscle of the participant with MELAS and a healthy control are displayed in Figure 1B. After the training period, the participant with MELAS exhibited an increase in MVC (5.5 N⋅m), EET (2.1 N⋅m) and VMA (13.1%). Interestingly, by normalizing MVC and EET for the thigh muscle mass all PRE to POST differences were conserved (Table 2).

**Table 2:** Effects of resistance training (RT) on body composition, muscle function, *in vivo* and *in vitro* mitochondrial respiration capacity in a subject with MELAS.

	MELAS		CTRL
		
	PRE	POST	
**Body composition**			
Body mass (kg)	36.2	37.6	70.7 ± 5.1
Body fat (%)	30.5	30.8	11.9 ± 2.5
Thigh muscle mass (kg)	2.11	2.21	6.85 ± 0.58
**Muscle function during 6 repetitions of isometric LE**			
MVC (Nm)	48.8 ± 4.4	54.3 ± 1.1	173 ± 15.4
EET (Nm)	11.6 ± 1.5	13.7 ± 0.9	41 ± 6.3
nMVC (Nm⋅kg^-1^)	23.1 ± 2.1	24.6 ± 0.5	25.2 ± 0.8
nEET (Nm⋅kg^-1^)	5.5 ± 0.7	6.2 ± 0.4	6.4 ± 0.5
VMA (%)	68.2 ± 7.9	81.3 ± 4.9	94.2 ± 3.1
**In vivo mitochondrial respiration capacity**			
End exercise mVO_2_ (mM⋅min^-1^)	1.99	2.01	9.82 ± 3.3
Δ mVO_2_ (mM⋅min^-1^)	1.71	1.79	8.61 ± 2.5
*τ* mVO_2_ (s)	94.7	24.5	21.3 ± 3.2


*Data of the control participants (CTRL) and muscle function values are given as mean ± standard deviation. MELAS, Mitochondrial encephalomyopathy, lactic acidosis, and recurrent stroke-like episodes syndrome; MVC, maximal voluntary contraction; EET, electrical evoked twitch; nMVC, maximal voluntary contraction normalized by thigh muscle mass; nEET, electrical evoked twitch normalized by thigh muscle mass; VMA, voluntary activation; mVO_*2*_, muscle oxygen consumption.*

### *In vivo* Muscular Mitochondrial Respiration Capacity

**FIGURE 2:**
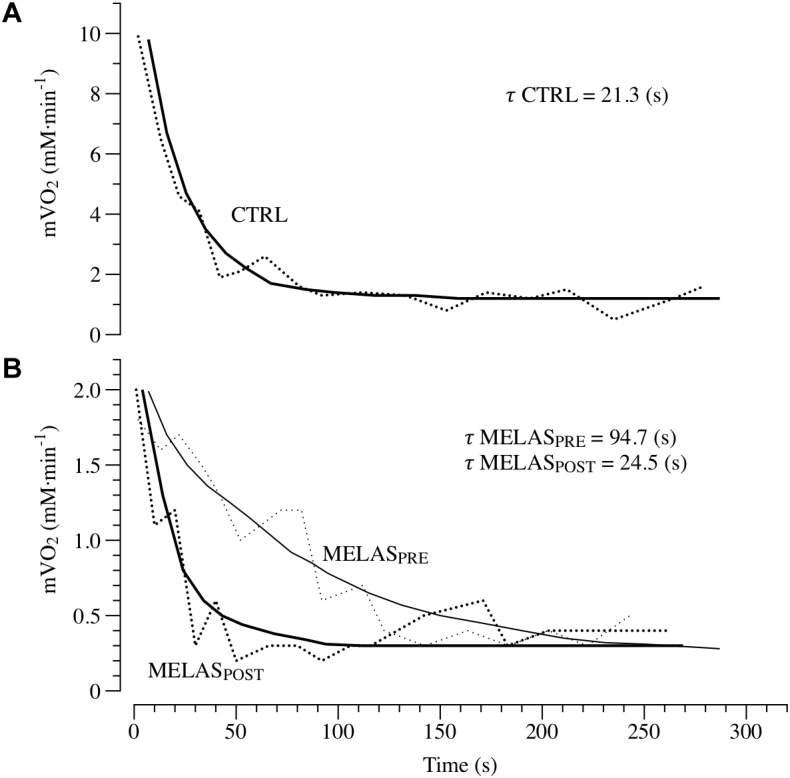
*In vivo* mitochondrial respiration capacity. Panels **(A,B)** presents examples of the oxidative capacity measured by NIRS in the plantar flexor of a healthy 21-year-old male (CTRL) and a patient with MELAS before (MELAS_PRE_) and after (MELAS_POST_) 12 weeks of resistance training. The mVO_2_ recovery data are fit to an exponential (continuous lines) to estimate the recovery *k*. The time constant (τ) is the reciprocal of the rate constant *k* (τ = 1/k).

Representative tracing of mVO_2_ kinetics in a healthy control and the participant with MELAS are displayed in Figure 2, Panels A and B, respectively. As illustrated in Table 2 after 12 weeks of RT the participant with MELAS exhibited no detectable changes in the end-exercise mVO_2_ (0.02 mM min^-1^), Δ mVO_2_ (0.09 mM min^-1^), while there was a marked amelioration in the *τ* mVO_2_ (Δ70.2 s).

## Discussion

Although RT has been shown to be feasible and likely reduce some effects of skeletal muscle disuse in patients with mitochondrial dysfunction, the effects of RT on the neuromuscular and mitochondrial function in MELAS are not established. In the present study we investigated the effects of RT on skeletal muscle and mitochondrial function in a 21 years old male patient with MELAS. In accordance with our hypothesis the main findings of this study were: (1) After the training period the locomotor-limb function was significant increased, exhibiting strong ameliorations in MVC (5.5 N⋅m), EET (2.1 N⋅m) and (2) These ameliorations in muscle function were coupled with structural gains of muscle mass in the locomotor limbs (13%) and a significant improvement of muscle recruitment, VMA (13%). Interestingly, the *in vivo* muscular respiratory capacity (*τ* mVO_2_) was also partially ameliorated after the RT. These promising results suggest that RT is an effective exercise approach in order to improve neurophysiological factors and muscles mass, in patients with MELAS.

### Structural, Neuromuscular and Muscle Respiratory Capacity in MELAS

As expected, before the exercise training the muscle mass of locomotor limbs was extremely reduced in the patient with MELAS as indicated by the limited thigh muscle mass (MELAS: 2.11 kg; CTRL: 6.85 ± 0.58). This limitation of skeletal muscle mass was coupled with a severe reduction of MVC (MELAS: 48.8 ± 4.4 Nm; CTRL: 173 ± 15.4 Nm), and EET (MELAS: 11.6 ± 1.5 Nm; CTRL: 41 ± 6.3 Nm). Moreover, maximal voluntary activation was severely reduced (MELAS: 68.2 ± 7.9%; CTRL: 94.2 ± 3.1%). Overall these results are comparable of those obtained in oldest-old and mobility limited individuals (Venturelli et al., 2015, 2018), suggesting that this reduction of muscle mass and force was partially due to the direct effect of MELAS and the partial disuse of the locomotor muscles. Moreover, muscle respiratory capacity was substantially lower in the patient with MELAS as indicated by the slower mVO_2_ time constant (MELAS: 94 s; Controls = 21.3 ± 3.2). In agreement with this result, a twofold slower PCr recovery time constant measured by ^31^P Magnetic Resonance Spectroscopy has previously been documented in a female patient suffering of MELAS compared to controls (Szendroedi et al., 2009). Compared with other studies examining muscle respiratory capacity *in vivo* in various patients’ populations, the value reported here in this individual with MELAS are also on the lower end of the spectrum. For instance, faster Time Constant (∼30–60 s) assessed by NIRS has been documented in the lower limb muscle of patients with Cystic Fibrosis (Erickson et al., 2015), Amyotrophic Lateral Sclerosis (Ryan et al., 2014b), and Multiple Sclerosis (Harp et al., 2016). In fact, the value observed here in our patient is similar to those reported in individuals with Spinal Cord Injury [∼85 s, (Erickson et al., 2017)], which due to the denervation of the muscle exhibit an extreme level of deconditioning and severe muscle atrophy. Together, these findings suggest severe functional abnormalities of the skeletal muscle mitochondria, which would explain the high susceptibility to the development of type 2 diabetes in these patients (Velho et al., 1996;Becker et al., 2002; Stark and Roden, 2007).

### Neuromuscular and Structural Effects of RT

Previously, Taivassalo et al., 1998, 1999, 2006 and Taivassalo and Haller (2004, 2005) and lately Murphy et al. (2008) reported a clear physiological rationale for the implementation of RT in the standard rehabilitation program for patients with mitochondrial dysfunctions. Specifically, these studies revealed a significant increase in leg muscle strength following RT (Murphy et al., 2008) and the results of the current study are in agreement with these previous investigations. Moreover, the present findings advance the knowledge on the interactions between neuromuscular and structural adaptations in response to RT in a rare pathology such the MELAS. The data from the current investigation indicate that RT generated positive effects on muscle structure and function. Specifically, we observed an increase in both neuromuscular activation and muscle mass of the limbs interested by the training (Table 2 and Figure 1). Moreover, the nMVC, nEET, and VMA indexes, suggests that after RT the gain of force was primarily determined by ameliorations of the neuromuscular function rather than hyper-trophy of the skeletal muscle. This RT-induced amelioration in the neuromuscular function was likely associated by changes in cortical function such as decrease in inhibition and increased activity in many areas of the cerebral cortex. Moreover, it is reasonable to assume that RT played a significant role in the amelioration of voluntary force production in the participant with MELAS likely due to greater intra and intermuscular recruitment.

### Effects of RT on Mitochondrial Respiration

It is well established that RT increases skeletal muscle force through the interplay of neuromuscular adaptations and the increase in cross-sectional area of the muscle. However, recent evidence suggest that mitochondrial adaptations can also occur (Murphy et al., 2008). Specifically, Murphy et al. (2008) revealed RT-related amelioration of VO_2_peak, coupled with decreased in COX-deficient cells and increased COX activity, suggestive of improved mitochondrial function within skeletal muscle (Taivassalo et al., 2001; Taivassalo and Haller, 2004, 2005). Interestingly, short-term RT has also been reported to increase skeletal muscle respiratory capacity measured *in vitro* and *in vivo* in young and older individuals (Jubrias et al., 2001; Pesta et al., 2011). The results of the current study give further credence to the hypothesis that RT can be a potent stimulus to induce mitochondrial adaptations in healthy populations and patients with MELAS. As illustrated in Figure 2, the time constant of mVO_2_ was drastically shortened from ∼94 s at baseline to ∼25 s post training, i.e., a value similar to the control group (∼21 s). Given the impairment in glucose control and severe exercise intolerance associated with this disease, these findings provide a proof of concept that RT represent a safe and effective training method to induce metabolic adaptations in the skeletal muscle of patients with MELAS. The *in vivo* approach used herein to measure muscle respiratory capacity with NIRS assess the integrated function of muscle O_2_ transport and utilization. Therefore, while it is unlikely that improvements in the convective or diffusive components of O_2_ delivery occurred with this type of training, this possibility cannot be entirely ruled out. Based upon previous studies reporting both an increase in mitochondrial content and function (Jubrias et al., 2001; Pesta et al., 2011), and given the magnitude of the change in muscle respiratory capacity observed here, it is likely that both structural and functional changes contributed to this improvement. Further studies using *in vitro* measurements of mitochondrial content and respiratory function are therefore warranted to clarify the exact mechanism contributing to this improvement in muscle respiratory capacity *in vivo*. It is important to note, that the muscle metabolic abnormalities observed in the patient with MELAS are likely due to the disuse of locomotor limbs but also primarily affected by an intrinsic metabolic problem related to MELAS, and unfortunately partially corrigible with increased physical activity. This result is partially in contrast with previous investigation reporting the disappearance of neuromuscular (Gruet et al., 2016) or metabolic (Decorte et al., 2017) abnormalities in physically active patients with cystic fibrosis.

## Conclusion

To conclude, this study suggests a preserved plasticity in the skeletal muscle of a patient with MELAS. More importantly, Resistance Training appears to be a safe and effective method to increase skeletal muscle function in this patient population, and this effect is mediated by both neuromuscular and mitochondrial adaptations. However, particular attention and caution is needed in the interpretation of the data of this single case study and further studies are warranted including larger sample of patients.

## Ethics Statement

For this case study the participant caregiver provided written informed consent.

## Author Contributions

MV and FS had full access to all of the data in the study and take responsibility for the integrity of the data and the accuracy of the data analysis. MV, FS, FV, FR, and DR designed and conducted the study. MV, FV, SS, CF, AP, NS, GL, EC, CM, and VC collected, analyzed, and interpreted the data. All authors reviewed and approved the final manuscript.

## Conflict of Interest Statement

The authors declare that the research was conducted in the absence of any commercial or financial relationships that could be construed as a potential conflict of interest.
